# Effectiveness of the kakaritsuke-yakuzaishi (family pharmacist) system for underage individuals in Japan: a cohort study using a health insurance claims database

**DOI:** 10.1186/s12913-025-13358-5

**Published:** 2025-09-02

**Authors:** Ryo Iketani

**Affiliations:** 1 Division of Pharmacoepidemiology, Showa Medical University Graduate School of Pharmacy, 1-5-8 Hatanodai, Shinagawa-ku, Tokyo, 142-8555, Japan; 2 Center for Outcomes Research and Economic Evaluation for Health, National Institute of Public Health, 2-3-6 Minami, Wako-shi, Saitama, 351-0197, Japan

**Keywords:** Additive interaction, Administrative claims data, Community pharmacy, Pharmacist, Retrospective cohort study

## Abstract

**Background:**

The kakaritsuke-yakuzaishi system (hereafter called the family pharmacist system), which provides more pharmaceutical services in exchange for higher fees than do general pharmacy practices, was introduced in Japan in April 2016. This cohort study aimed to describe the characteristics of patients who used the family pharmacist system and assess its effectiveness in the pharmaceutical management of underage individuals. It also evaluated the effect modifications of age, number of types of drugs, and number of medical facilities used on the effectiveness of the system.

**Methods:**

This study comprised underage individuals who visited pharmacies every six months between April 2017 and March 2020 from the Japan Medical Data Centre health insurance database, categorizing them as users and non-users of the family pharmacist system. The claim rates (per 1,000 person-visits) of fees for adjusting leftover drugs and preventing therapeutic duplication and drug interactions (TDDIs) were calculated as endpoints. Group comparisons were performed by applying a generalized estimation equation to groups whose characteristic variables were balanced by the inverse probability of treatment weighting. The effect modifications on the endpoints were assessed based on the relative excess risk due to interaction (RERI) of the candidate variables.

**Results:**

The eligible cohort comprised 200,673 underage patients (users: 6,109; non-users: 194,564). Users tended to be younger patients, who received prescriptions for more drug types and used more medical facilities than non-users did. Regarding the fee for adjusting leftover drugs, the claim rates were 0.6 for users and 0.4 for non-users (incidence rate ratio [IRR]: 1.6; 95% confidence interval [CI]: 0.9–2.9). Regarding the fee for preventing TDDIs, the claim rates were 4.8 for users and 3.7 for non-users (IRR: 1.3; 95% CI: 1.1–1.4). No effect modification was detected in RERI.

**Conclusions:**

This study demonstrated that the family pharmacist system improved TDDI prevention among underage individuals. However, its effectiveness was consistent regardless of the characteristics related to the utilization of the family pharmacist system, indicating that the trend of positively applying the system to those with these characteristics was not supported. The operation of the system may be reconsidered for the efficient allocation of medical resources.

**Supplementary Information:**

The online version contains supplementary material available at 10.1186/s12913-025-13358-5.

## Background

The Japanese health system is based on a fee-for-service reimbursement according to the national medical fee schedule [1]. Medical services provided by pharmacies also adhere to this medical fee schedule, which outlines the fees for dispensing and individual pharmacotherapy management [2, 3]. This schedule is reviewed every two years to control the total medical costs and facilitate specific medical procedures. The kakaritsuke-yakuzaishi system (hereafter called the family pharmacist system) was established in April 2016 by including kakaritsuke-yakuzaishi-shidouryo (hereafter referred to as the family pharmacist consultation fee) in the schedule to facilitate centralized and consistent pharmaceutical care [2]. The operation of the family pharmacist system begins when a particular pharmacist obtains consent from a patient to claim a family pharmacist consultation fee that is higher than the fee for general pharmaceutical management. Family pharmacists are required to provide pharmaceutical services, including 24-h availability for consultations, tracking dispensing information from other pharmacies, and gathering feedback from physicians. Although these services may overlap with general pharmaceutical management, the system differs in that it clearly specifies the conditions for claiming fees. The family pharmacist system can be regarded as a system that requires a higher fee in exchange for more medical services in pharmacies.

Several studies have explored the effectiveness of this system in adults [4, 5]. These studies demonstrated an association between its use and an increase in claims for choufukutouyaku-sougosayoutou-boushi-kasan (hereafter called the therapeutic duplication and drug interaction [TDDI] prevention fee). This fee can be claimed when prescriptions are changed to prevent TDDI or to adjust for leftover drugs. Thus, the family pharmacist system and incentives for concentrated pharmaceutical management facilitate better pharmacotherapy for adult users than for non-users.

However, the effectiveness and practical implementation of this system in underage populations (defined as individuals aged < 18 years) remain unclear. The effectiveness of the system possibly varies owing to differences in disease structure between adults and underage individuals, which affect pharmaceutical management practices. A previous study showed that there were fewer pharmaceutical interventions for underage populations during admission than for adult populations [6]. Fewer conditions require concentrated pharmaceutical management in underage individuals. Differences in the need for pharmaceutical management influence the effectiveness of the family pharmacist system among the applied populations. In addition, several studies have reported a relationship between subsidies for underage individuals and increases in health service utilization [7–10]. In Japan, concerns have been raised that subsidies for underage individuals could lead to the unnecessary utilization of medical services and increased medical costs. This trend may be pronounced in the population aged < 6 years, as they benefit from subsidies and low out-of-pocket payments [1, 7–10]. If the family pharmacist system is used more for this population nevertheless the system’s usefulness is homogeneous among the overall underage population, its operation would then need to be modified to allocate medical resources appropriately. Thus, to design efficient policies, it is crucial to retrospectively evaluate whether individual medical policies should target specific population groups, as accessibility to medical care differs among populations.

Therefore, this cohort study aims to examine the effectiveness of the family pharmacist system in underage patients and assess the heterogeneity of its effectiveness using administrative claims data.

## Methods

**Fig. 1:**
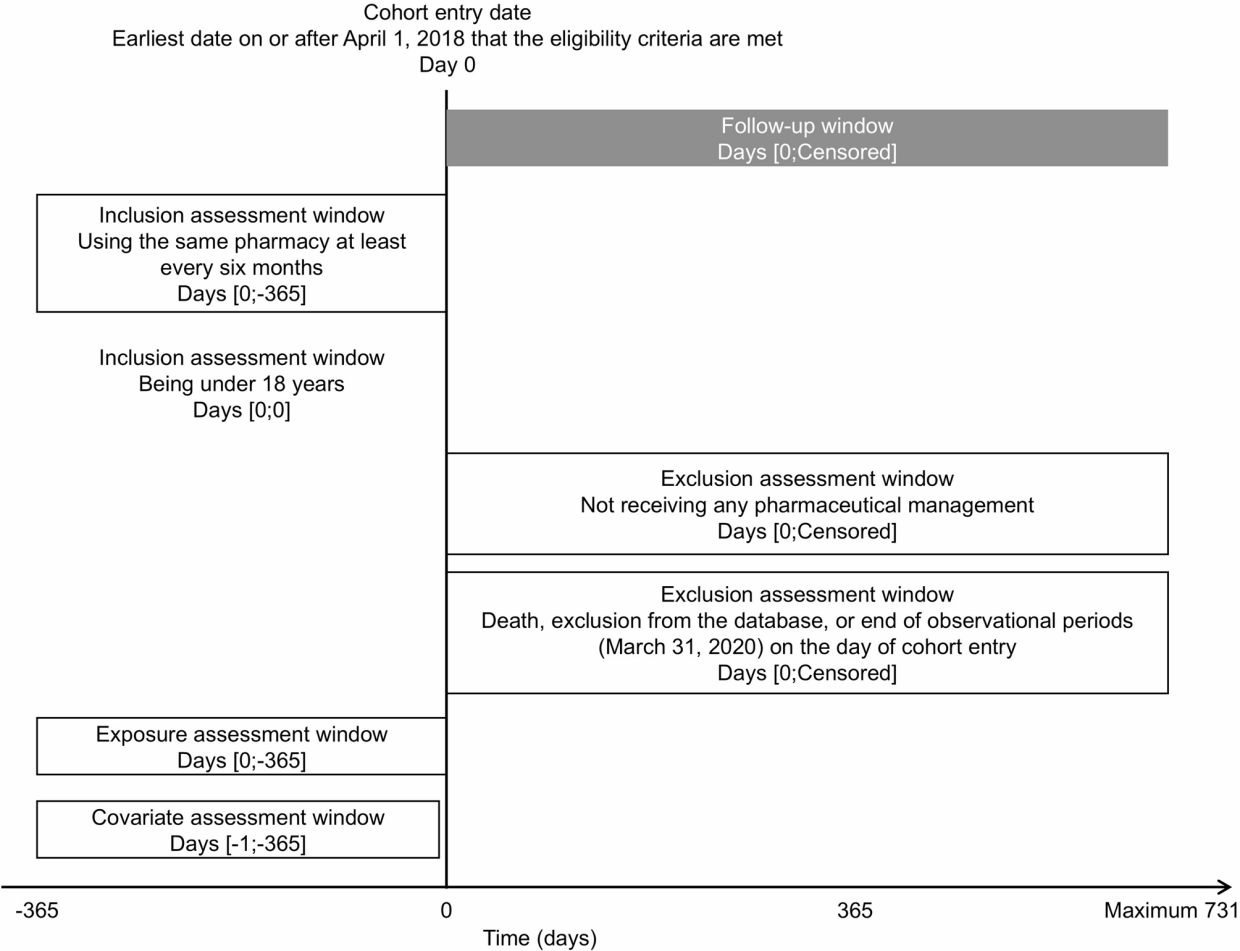
Design diagram of the study. The cohort considering group switching followed patients from cohort entry until censoring, which was defined as death, exclusion from the database, group switching, or March 31, 2020. The observation period ranged from a minimum of two to a maximum of 731 days

The study design diagram is shown in Fig. 1 [11].

### Data source and cohort definition

This study used data from the Japan Medical Data Centre health insurance database, which includes administrative claims data (medicine, diagnostic payment combination, and dispensing) and standard demographic information (birth year and month, sex, and enrollment period). The details of this database have been described previously [12]. As of January 2021, it contained records for 11,065,454 individuals. Owing to data availability, 1.7 million individuals were randomly sampled from the entire cohort, and their data between April 2017 and March 2020 were used. The dynamic cohort comprised patients who used the same pharmacy at least every six months within one year before cohort entry and were aged < 18 years at cohort entry. This study cohort was limited to patients who used pharmacies continuously because the family pharmacist consultation fee can be claimed for such patients. Participants were categorized into user or non-user groups. The user group had a history of claiming a family pharmacist consultation fee at or before cohort entry. The non-user group had no history of claiming a family pharmacist consultation fee but received general pharmaceutical management at or before cohort entry. Classification based on a single claim for the family pharmacist consultation fee enabled estimation of the intention-to-treat effect. This estimand was important, as the impact of the family pharmacist system may extend to pharmaceutical care at pharmacies other than the designated family pharmacy. Exclusion criteria were as follows: not receiving any pharmaceutical management during the observation period and death, exclusion from the database, or end of the observation period (March 31, 2020) on the day of cohort entry. The observation period was from cohort entry until death, exclusion from the database, occurrence of endpoints, or March 31, 2020. This study considered group changes during the observation period. Participants who were claimed for the family pharmacist consultation fee after cohort entry were followed up as the non-user group until the point of the initial claim and subsequently as the user group.

### Endpoints

Two types of TDDI prevention fees are defined: one for prescription changes attributable to leftover drugs, and another for prescription changes to prevent TDDI. This study evaluated claims for these fee types during the observation period. These claims directly reflect the results of pharmaceutical interventions and enable result comparisons between the underage and adult populations.

### Covariates

The following covariates were collected within six months before cohort entry or group changes for both groups: age, sex, number of types of drugs, number of medical institutions used, use of multiple hospital departments, number of medical examinations, admission, and prescribed drugs (cold medicine, non-steroidal anti-inflammatory drugs, acetaminophen, antibiotics, H1 blockers, asthma inhalers, tulobuterol, leukotriene receptor antagonists, probiotics, laxatives, prokinetics, steroids, antiepileptic drugs, traditional Japanese herbal medicines, skin barriers, heparinoids, and topical steroids). Age, number of types of drugs, and number of medical institutions used were categorized at clinically meaningful levels. The definitions of these variables are presented in eTable 1 of Additional File 1. All covariates were available for all participants.

### Propensity score weighting

Based on the collected 24 covariates and age-squared term, propensity scores (PSs) for users and non-users of the family pharmacist system were estimated using a multivariable logistic regression model. The stabilized weights were calculated using the estimated PSs and applied to the study cohort to balance the distribution of covariates between groups [13]. The balance of the covariates was assessed based on whether the absolute value of the standardized mean difference (SMD) was < 0.100 [13].

### Statistical analysis

Continuous variables are described as means (standard deviations [SDs]), and categorical variables are described as frequencies (%).

The claim frequency, proportion (%), and rate (per 1,000 person-visits) of TDDI prevention fees were calculated. The unweighted and weighted Kaplan–Meier curves are depicted by groups. A generalized estimation equation (GEE) with a Poisson distribution and log link function was used to estimate incidence rate ratios (IRRs) and 95% confidence intervals (CIs), using the sandwich estimator to assess the association between groups and endpoints. This model included the number of pharmacy visits during the observation period as an offset term. The statistical significance of the IRRs was determined based on whether the 95% CIs reached 1.0.

The effect modification is evaluated on an additive or multiplicative scale. Evaluating the effect modification of the additive scale explains the allocation of medical resources. The relative excess risk due to interaction (RERI) is an index that enables the evaluation of the additive effect modification from the effect measure on a ratio scale [14]. The RERIs were calculated for age (< 6 vs. ≥ 6) and the use of the family pharmacist system, based on IRRs estimated using a GEE model that included the interaction term and the individual variables [14, 15]. Similarly, the additive effect modifications by the number of types of prescribed drugs (≥ 6 vs. < 6) and the number of medical facilities used (≥ 2 vs. 1) were subjected to this evaluation, as patients with these factors were considered candidates for the family pharmacist system [5]. Positive RERIs indicate that the claims for the TDDI prevention fee were facilitated by the user who had the characteristics of age < 6 years, with prescription ≥ 6 types of drugs, and using ≥ 2 medical facilities. Negative RERIs indicate an inverse relationship with positive RERIs. The 95% CIs for the RERIs were calculated using the delta method [14]. The statistical significance of the RERIs was determined based on whether the 95% CIs reached 0.

### Sensitivity analysis

The following sensitivity analyses were performed: (I) weighting the cohort using the PSs where the > 95th percentiles were trimmed to further eliminate the impact of extreme weights; (II) limiting patients to those using a single pharmacy during the observation period to enhance the consistency of pharmaceutical management; (III) limiting patients to those using pharmacies with a history of claiming the family pharmacist consultation fee to make the pharmacy feature homogeneous; and (IV) limiting patients to those not claiming the TDDI prevention fee during the pre-observation periods to evaluate the impact of previous pharmaceutical intervention.

## Results

### Eligible cohort

**Fig. 2:**
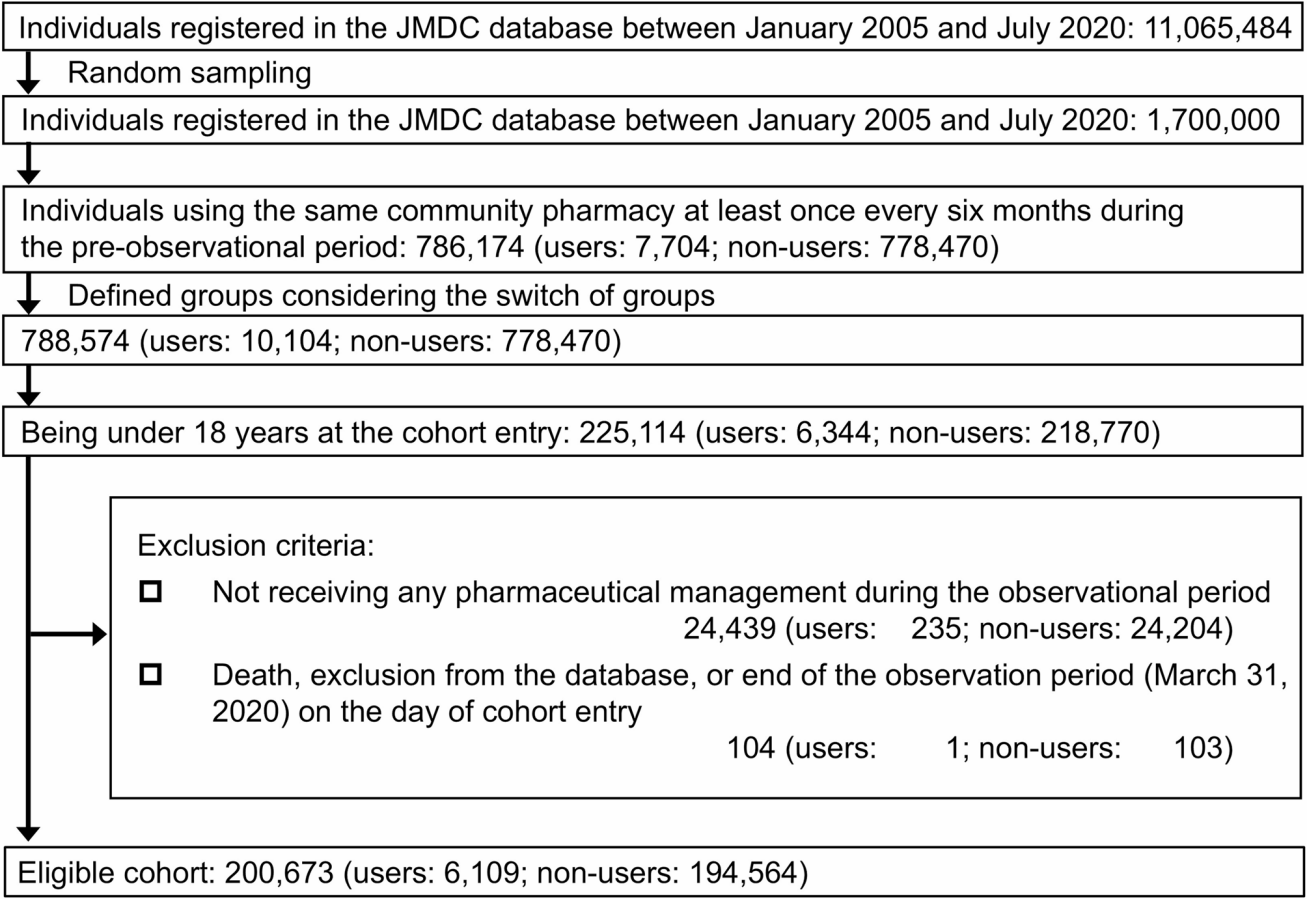
Flow diagram of the study. Abbreviation: *JMDC* Japan Medical Data Centre

Considering group switches, the eligible cohort comprised 200,673 underage patients (users: 6,109; non-users: 194,564) (Fig. 2). The mean ages (SDs) were 5.0 (3.9) years for users, 7.3 (5.1) years for non-users, and 7.2 (5.1) years for the overall population. The user group included a higher proportion of patients aged < 6 years than did the non-user group (users: 61.8%; non-users: 42.4%) (Table 1). The user group received prescriptions for more types of drugs, visited more medical institutions, and visited hospitals more frequently than the non-user group did.

**Table 1:** Clinical characteristics before and after the inverse probability of treatment weighting

Variables	Before weighting			After weighting		
	User (*N* = 6,109)	Non-user (*N* = 194,564)	SMD	User (*N* = 6,144)	Non-user (*N* = 194,566)	SMD
Age, years	5.0 (3.9)	7.3 (5.1)	−0.494	7.4 (5.2)	7.2 (5.1)	0.032
< 6	3,776 (61.8)	82,457 (42.4)	0.397	2,675 (43.5)	83,516 (42.9)	0.012
Sex						
Male	3,271 (53.5)	100,246 (51.5)	0.040	3,125 (50.9)	100,368 (51.6)	−0.014
Female	2,838 (46.5)	94,318 (48.5)	−0.040	3,019 (49.1)	94,198 (48.4)	0.014
Number of types of drugs	11.7 (5.5)	9.5 (5.0)	0.416	9.5 (5.0)	9.6 (5.1)	−0.003
≥ 6	5,363 (87.8)	149,011 (76.6)	0.296	4,752 (77.3)	149,632 (76.9)	0.010
Number of medical institutions used	2.7 (1.4)	2.3 (1.2)	0.275	2.4 (1.3)	2.4 (1.2)	−0.007
≥ 2	4,890 (80.0)	140,242 (72.1)	0.188	4,373 (71.2)	140,745 (72.3)	−0.026
Use of multiple departments in a hospital	115 (1.9)	2,248 (1.2)	0.059	79 (1.3)	2,292 (1.2)	0.010
Number of medical examinations	9.1 (6.9)	6.7 (5.8)	0.374	7.0 (5.7)	6.8 (5.9)	0.036
Admission	264 (4.3)	5,439 (2.8)	0.082	193 (3.1)	5,531 (2.8)	0.017
Concomitant drugs						
Cold medicines (cough medicines, expectorants, general cold medicines)	5,377 (88.0)	148,264 (76.2)	0.312	4,588 (74.7)	148,965 (76.6)	−0.044
NSAIDs	540 (8.8)	27,103 (13.9)	−0.161	827 (13.5)	26,801 (13.8)	−0.009
Acetaminophen	3,948 (64.6)	111,880 (57.5)	0.146	3,413 (55.5)	112,302 (57.7)	−0.044
Antibiotics	3,803 (62.3)	107,664 (55.3)	0.141	3,334 (54.3)	108,075 (55.5)	−0.026
H1 blockers	4,349 (71.2)	116,948 (60.1)	0.235	3,654 (59.5)	117,607 (60.4)	−0.020
Asthma inhalers	762 (12.5)	15,781 (8.1)	0.144	552 (9.0)	16,041 (8.2)	0.026
Tulobuterol	2,316 (37.9)	47,605 (24.5)	0.293	1,486 (24.2)	48,403 (24.9)	−0.016
Leukotriene receptor antagonists	2,549 (41.7)	60,223 (31.0)	0.225	1,971 (32.1)	60,865 (31.3)	0.017
Probiotics	3,349 (54.8)	77,654 (39.9)	0.302	2,464 (40.1)	78,541 (40.4)	−0.005
Laxatives	358 (5.9)	7,569 (3.9)	0.092	247 (4.0)	7,687 (4.0)	0.003
Prokinetics	1,067 (17.5)	25,402 (13.1)	0.123	829 (13.5)	25,665 (13.2)	0.009
Steroids	480 (7.9)	11,412 (5.9)	0.079	383 (6.2)	11,531 (5.9)	0.013
Antiepileptic drugs	113 (1.8)	1,602 (0.8)	0.089	80 (1.3)	1,665 (0.9)	0.044
Traditional Japanese herbal medicines	498 (8.2)	14,350 (7.4)	0.029	489 (8.0)	14,398 (7.4)	0.021
Skin barriers	1,695 (27.7)	40,905 (21.0)	0.157	1,281 (20.9)	41,305 (21.2)	−0.009
Heparinoid	2,428 (39.7)	57,298 (29.4)	0.218	1,815 (29.5)	57,910 (29.8)	−0.005
Topical steroids	3,189 (52.2)	81,042 (41.7)	0.213	2,558 (41.6)	81,669 (42.0)	−0.007

Continuous and categorical variables are summarized as mean (standard deviation) and frequency (%), respectively. An absolute value of SMD < 0.100 indicates that the covariates were balanced between the groups

Abbreviations: *NSAIDs* Non-steroidal anti-inflammatory drugs, *SMD* Standardized mean difference

The cohort was weighted using PSs, resulting in balanced covariate distributions between the groups, as indicated by the SMDs for all covariates (< 0.100).

### Adjustment of leftover drugs

**Fig. 3:**
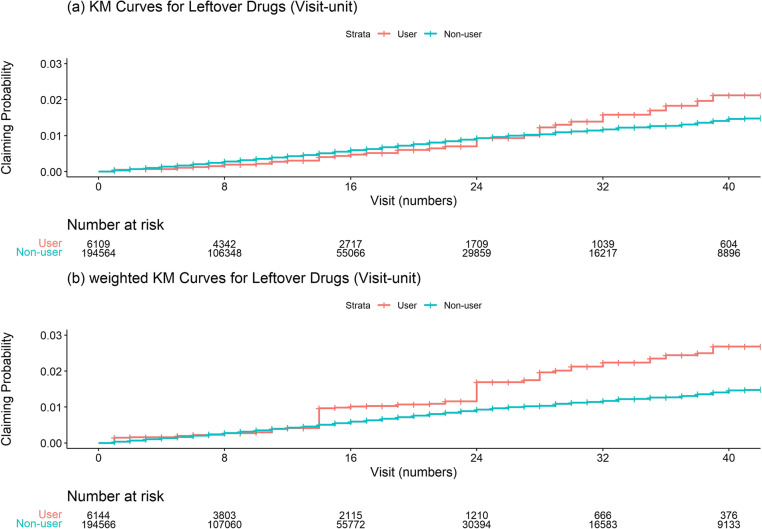
Unweighted and weighted Kaplan–Meier curves for leftover drugs. Abbreviation: *KM* Kaplan-Meier

In the weighted cohort, the claim rates were 0.6/1,000 person-visits in the user group and 0.4/1,000 person-visits in the non-user group (IRR: 1.6; 95% CI: 0.9–2.9) (Table 2; Fig. 3). Sensitivity analyses showed that the point estimates of the IRRs ranged from 1.3 to 2.4. All analyses indicated a superior tendency among users but showed no statistical significance.

**Table 2:** Comparison of the endpoints between the groups

	User		Non-user		**IRR (95% CI)**
**Adjustment of leftover drugs**	**Events/** *N* ** (%)**	**Rate**	**Events/** *N* ** (%)**	**Rate**	
Before weighting	47/6,109 (0.8)	0.4	896/194,564 (0.5)	0.4	1.2 (0.9 to 1.6)
After weighting	53/6,144 (0.9)	0.6	903/194,566 (0.5)	0.4	1.6 (0.9 to 2.9)
Sensitivity analysis I (Trimming)	53/6,144 (0.9)	0.6	902/194,515 (0.5)	0.4	1.6 (0.97 to 2.7)
Sensitivity analysis II (Single pharmacy)	6/1,095 (0.6)	0.9	77/43,031 (0.2)	0.4	2.4 (0.7 to 7.8)
Sensitivity analysis III (Family pharmacy)	46/4,959 (0.9)	0.5	221/36,313 (0.6)	0.4	1.3 (0.8 to 2.2)
Sensitivity analysis IV (Without event)	49/5,881 (0.8)	0.6	845/190,864 (0.4)	0.3	1.6 (0.9 to 2.9)
**Prevention of TDDI**	**Events/N (%)**	**Rate**	**Events/N (%)**	**Rate**	**IRR (95% CI)**
Before weighting	523/6,109 (8.6)	5.0	8,877/194,564 (4.6)	3.7	1.3 (1.2 to 1.5)
After weighting	408/6,144 (6.6)	4.8	8,982/194,566 (4.6)	3.7	1.3 (1.1 to 1.4)
Sensitivity analysis I (Trimming)	411/6,144 (6.7)	4.8	8,973/194,515 (4.6)	3.7	1.3 (1.1 to 1.4)
Sensitivity analysis II (Single pharmacy)	42/1,095 (3.8)	6.3	649/43,031 (1.5)	3.3	1.9 (1.3 to 2.9)
Sensitivity analysis III (Family pharmacy)	388/4,959 (7.8)	4.8	2,337/36,313 (6.4)	4.5	1.1 (0.95 to 1.2)
Sensitivity analysis IV (Without event)	350/5,881 (6.0)	4.3	8,325/190,864 (4.4)	3.6	1.2 (1.1 to 1.4)

The rate indicates the frequency per 1,000 person-visits

Abbreviations: *CI* Confidence interval, *IRR* Incidence rate ratio, *TDDI*, therapeutic duplication and drug interaction

**Fig. 4:**
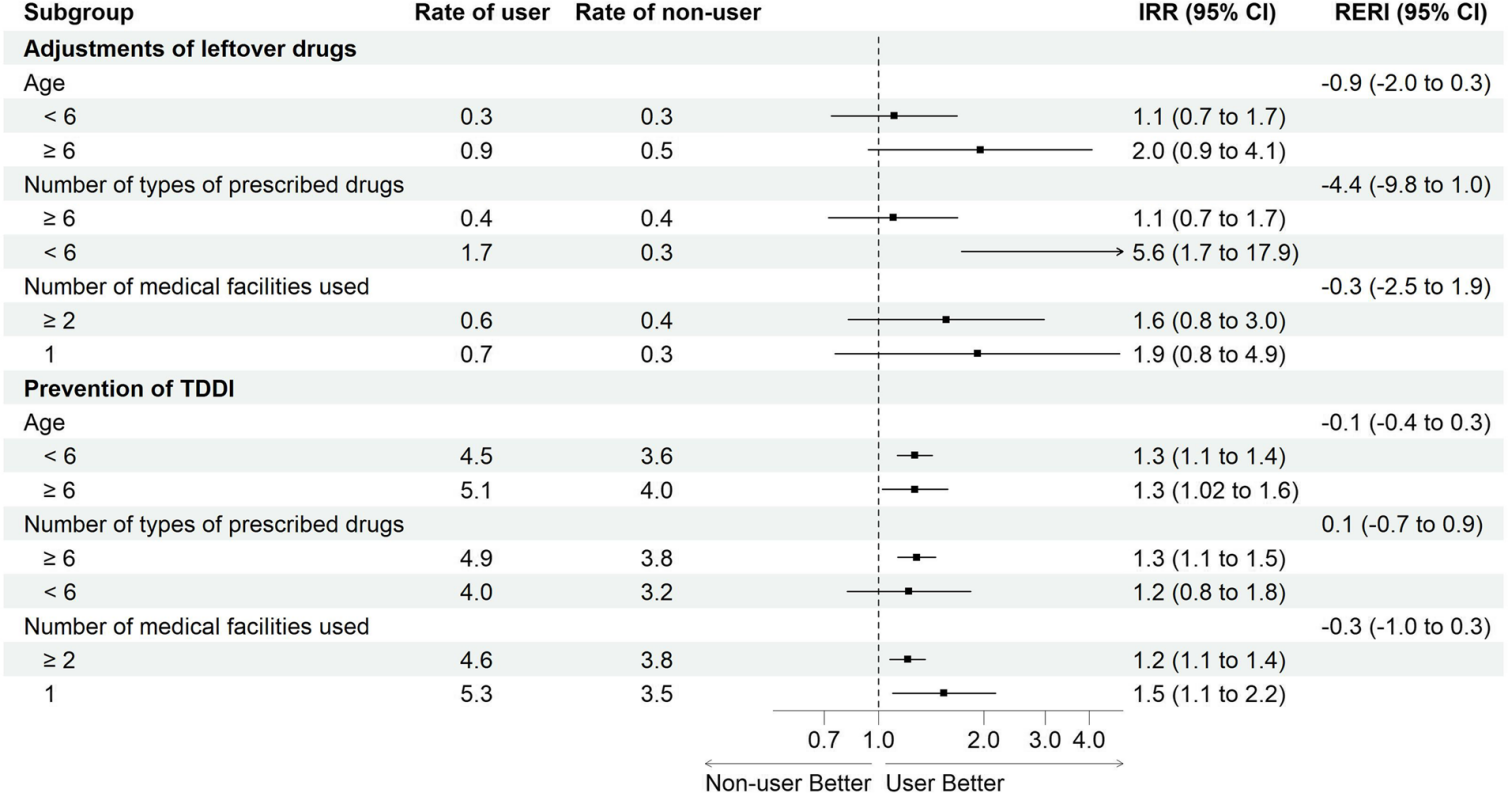
Effect modification of covariates on the effectiveness of the family pharmacist system. Abbreviations: *CI* Confidence interval,* IRR* Incidence rate ratio, *RERI* Relative excess risk due to interaction, *TDDI* Therapeutic duplication and drug interaction. The rate indicates the frequency per 1,000 person-visits. Positive RERIs indicate that the claims for the TDDI prevention fee were facilitated by the user who had the characteristics of age < 6 years, with prescription ≥ 6 types of drugs, and using ≥ 2 medical facilities. Negative RERIs indicate an inverse relationship with positive RERIs

The RERIs for age, the number of types of prescribed drugs, and the number of medical facilities used indicated no effect modification of the family pharmacist system (Fig. 4).

### Prevention of therapeutic duplication and drug interaction

**Fig. 5:**
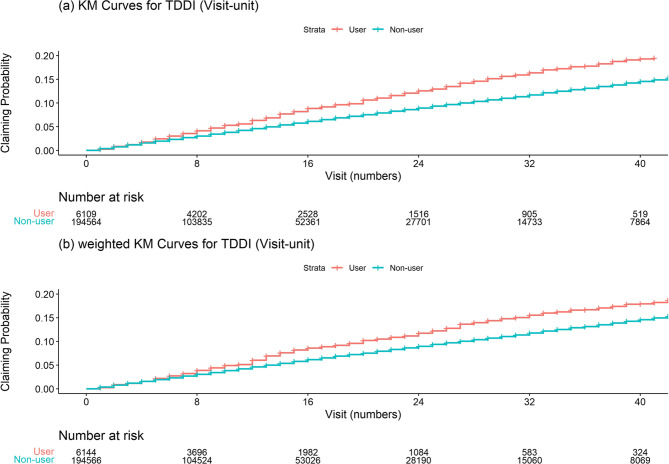
Unweighted and weighted Kaplan–Meier curves for TDDI. Abbreviations:*KM* Kaplan-Meier, *TDDI* Therapeutic duplication and drug interaction

In the weighted cohort, the claim rates were 4.8/1,000 person-visits in the user group and 3.7/1,000 person-visits in the non-user group (IRR: 1.3; 95% CI: 1.1–1.4) (Table 2; Fig. 5). Sensitivity analyses showed that the point estimates of the IRRs ranged from 1.1 to 1.9. All analyses indicated a superior tendency for users. Statistical significances were detected in all analyses except sensitivity analysis III.

The RERIs for age, the number of types of prescribed drugs, and the number of medical facilities used indicated no effect modification of the family pharmacist system (Fig. 4).

## Discussion

The findings of this study suggest that the use of the family pharmacist system among underage individuals increases TDDI prevention but does not lead to elevated adjustment of leftover drugs. The effectiveness of the system in underage individuals is limited to providing safer pharmaceutical treatments than those in adults [4, 5]. The differences in the disease structure between adults and underage individuals may explain this varying effectiveness. This study revealed a lower absolute claim rate for leftover drugs than for the prevention of TDDI, indicating fewer cases requiring adjustments for leftover drugs among underage individuals than among adults. This low incidence rate might contribute to the absence of statistical significance in this large-scale study. Most underage individuals in this study had prescription histories primarily comprising medications for acute illnesses, including cold medicines, antipyretic analgesics, or antiallergic drugs, which were not used continuously. Thus, the system may not be sufficiently effective in adjusting leftover drugs in underage individuals. Similarly, the leftover drugs could have been reduced owing to improved adherence of patients to the family pharmacist system [16]. A previous study reporting that careful explanation by pharmacists improved adherence in pediatric patients supports this perspective [17].

Although the comparison of TDDI prevention demonstrated the system’s effectiveness, sensitivity analysis III (limiting patients to those using pharmacies with a history of claiming a family pharmacist consultation fee) did not statistically support its effectiveness, despite the high statistical power based on the large sample size. This analysis aimed to account for differences in behavior related to claiming TDDI prevention fees between pharmacies implementing a family pharmacist system and those that do not. Thus, this sensitivity analysis enhances the comparability between groups. Based on this analysis, the effectiveness of the system in preventing TDDI may be limited; however, the point estimates from all analyses consistently supported the effectiveness of the system. In addition, a previous study suggested that incentives promote pharmaceutical services in community pharmacies [18]. The family pharmacist system, which involves higher medical payments than those of general pharmaceutical management systems, possibly facilitates pharmaceutical management. Notably, this study merely detected an increase in pharmacological interventions in underage individuals, albeit at a lower level than in adults, with high statistical power. The clinical significance of this difference depends on the types of avoided TDDIs and their consequences, which could not be measured in this study. The relatively small differences in TDDI prevention may reduce the cost-effectiveness of the family pharmacist system in underage individuals compared with adults.

The use of the family pharmacist system was more pronounced in the < 6-year-old group than in the ≥ 6-year-old group. This trend may be attributed to subsidies and lower out-of-pocket medical expenses in the < 6 years age group. Medical subsidies for children are correlated with the increased use of medical resources [7–10]. For the family pharmacist system, these factors may induce a pharmacist’s proposal to use the system, as well as patient and parent acceptance, beyond medical necessity. Although this study examined whether age modified the effectiveness of the system, the claim rate for the TDDI prevention fee did not vary between the < 6 and ≥ 6 years age groups. Moreover, this study showed that the effectiveness of the family pharmacist system did not vary among patients who were prescribed many medicines or who used multiple medical facilities. These patients, who are considered candidates for the system, previously demonstrated enhanced effectiveness in adults [5]. In an underage population, the system may not sufficiently demonstrate its usefulness in optimizing prescriptions. This study involved the use of administrative claims data, which are elaborate records of pharmaceutical services, to clarify the actual operation and benefits of the system. The actual operation in which the < 6 years age group used the system requires a reconsideration of medical resource allocation. Currently, there are no eligibility conditions based on a patient’s medical background for claiming a family pharmacist consultation fee. It may be essential to revise these conditions by, for example, limiting the scope of application to adults. To further assess the appropriateness of allocating pharmaceutical services to underage individuals, it is important to consider additional aspects, such as patient and parent satisfaction, which were not evaluated in the present study. A qualitative analysis based on a questionnaire will reveal the value of the family pharmacist system.

### Limitations

This study has some limitations. Unmeasured confounding factors could exist in the analyses. For example, the use of over-the-counter drugs might affect TDDIs and confound the relationship between the system and its effectiveness. In addition, this study did not capture patients with poor drug adherence who were also considered candidates for the family pharmacist system [2]. An imbalance in the distribution of these patients could complicate the evaluation of the system’s effectiveness. Social and geographical statuses could also be confounding factors. As previously mentioned, differences in perceptions of costs affect how individuals use healthcare services. Health literacy and parental communication behaviors are related to pharmaceutical management [19]. In Japan, the distribution of community pharmacists is skewed [20]. Patients in areas with few pharmacists may receive little pharmaceutical services. The available data for this study did not allow for the use of instrumental variable analysis or the inclusion of a negative control to evaluate the robustness of the findings. Moreover, claims data may not fully reflect real-world events. In some cases, pharmacies did not claim a TDDI prevention fee when prescriptions were changed to prevent TDDIs or to adjust leftover drugs. This misclassification could affect the estimation of system effectiveness. Challenges in defining the scope of the family pharmacist system’s impact influenced group classification and endpoint measurement. This study may overestimate the system’s effect, as endpoints observed at any pharmacy in the user group were counted as events within that group.

## Conclusions

This study demonstrates that the family pharmacist system for underage individuals increased TDDI prevention. However, its effectiveness remained unchanged among patients aged < 6 years and those aged ≥ 6 years, even though the former used the family pharmacist system more frequently. Concerning the allocation of medical resources, considering the types of underage patients who are candidates for the system is crucial. Future studies should evaluate other aspects of the system in addition to providing safer pharmaceutical treatments for the underage population.

## Supplementary Information

Supplementary Material 1

## Data Availability

The data that support the findings of this study are available from JMDC Inc., but restrictions apply to the availability of these data, which were used under license for the current study and are not publicly available. Data are, however, available from the authors upon request and with permission of JMDC Inc. In that case, the corresponding author should be contacted.

## Abbreviations

CI: Confidence interval
GEE: Generalized estimation equation
IRR: Incidence rate ratio
PS: Propensity score
RERI: Relative excess risk due to interaction
SD: Standard deviation
SMD: Standardized mean difference
TDDI: Therapeutic duplication and drug interaction
