# Supplementary material for: Effectiveness of the kakaritsuke-yakuzaishi (family pharmacist) system for underage individuals in Japan: a cohort study using a health insurance claims database
Source: BMC Health Serv Res. 2025 Sep 2;25:1182. doi: 10.1186/s12913-025-13358-5 (PMC12406381; doi:10.1186/s12913-025-13358-5)
Supplement: Supplementary file 1 — Supplementary Material 1 [file 12913_2025_13358_MOESM1_ESM.docx]

**Supplementary Table 1. The definitions of covariates**

| **Covariate** | **Definition** |
| --- | --- |
| Age | age at the cohort entry |
| Sex | NA |
| Number of types of drugs | the sum of drugs in different chemical subgroups based on WHO-ATC code |
| Number of medical institutions used | the sum of different identifiers of medical institutions |
| Use of multiple departments in a hospital | the claims of the fees for using multiple departments in a hospital |
| Number of medical examinations | the sum of different days when dispensing fees were calculated |
| Admission | the record of either admission or the claims of the fees for admission |
| Concomitant drugs |  |
| Cold medicines (cough medicines, expectorants, general cold medicines) | WHO-ATC code: R05- |
| NSAIDs | WHO-ATC code: M01A-, M02AA-, M02AC-, M02BG-, N02AE01, N02AJ13, N02AX02, N02BE71, or N02CA52 in oral formulation or external preparation |
| Acetaminophen | WHO-ATC code: N02BE01 |
| Antibiotics | WHO-ATC code: J01- in oral formulation |
| H1 blockers | WHO-ATC code: R06A- in oral formulation |
| Asthma inhalers | WHO-ATC code: R03A- in external preparation |
| Tulobuterol | WHO-ATC code: R03CC11 in patch formulation |
| Leukotriene receptor antagonists | WHO-ATC code: R03DC- |
| Probiotics | WHO-ATC code: A07F- |
| Laxatives | WHO-ATC code: A06- |
| Prokinetics | WHO-ATC code: A03FA- |
| Steroids | WHO-ATC code: H02- |
| Antiepileptic drugs | WHO-ATC code: N03AA-，N03AB-，N03AC-，N03AD-，N03AE，N03AF-，N03AG-，N03AX03，N03AX07，N03AX09，N03AX10，N03AX11，N03AX12，N03AX13，N03AX14，N03AX15，N03AX17，N03AX18, or N03AX22 |
| Traditional Japanese herbal medicines | WHO-ATC code: V03B1- |
| Skin barriers | WHO-ATC code: D02A- |
| Heparinoid | WHO-ATC code: C05BA01 |
| Topical steroids | WHO-ATC code: D07- |

The information was collected for six months before cohort entry.

Abbreviations: NSAIDs, non-steroidal anti-inflammatory drugs; NA, not applicable; WHO-ATC, world health organization-anatomical therapeutic chemical classification system.
